# Disadvantaged Students Utilize School Campus and Its Resources More Than Non-disadvantaged Students

**DOI:** 10.7759/cureus.46128

**Published:** 2023-09-28

**Authors:** Kian Habashi, Shaun Andersen, Deepal Patel, Genesis K Leon, Cynthia Lee, Edward Simanton

**Affiliations:** 1 Medical Education, Kirk Kerkorian School of Medicine at the University of Nevada, Las Vegas, Las Vegas, USA

**Keywords:** low-resource, disparity, education, covid-19, campus, socioeconomic disadvantage, underrepresented minority, first-generation, disadvantaged

## Abstract

Introduction

Disadvantaged populations were disproportionately affected by the COVID-19 pandemic, both in the medical and educational settings. Lower-income families often do not have a laptop/desktop computer, adequate internet connection, or a dedicated study space. This unfortunately contributed to poorer academic performance during distance learning. To combat this, the Kirk Kerkorian School of Medicine (KKSOM) did not close down campus during the pandemic. This study analyzes the utilization of campus and live Zoom lectures by KKSOM students and its impact on educational outcomes.

Methods

We sent an Institutional Review Board (IRB)-approved survey to KKSOM students and asked about study locations, Zoom lecture attendance, and relationship quality during the pandemic. The class of 2024 had a unique experience as they were first-year students during the start of the COVID-19 pandemic and the transition to online learning. However, they always had access to campus and technological resources. We compared the survey scores from a Qualtrics electronic survey and the National Board of Medical Examiners (NBME) scores of students who self-indicated disadvantaged status, first-generation, underrepresented minority, and lower socioeconomic status to those who did not meet these criteria. Data analysis was done using SPSS software version 28.0.1.1 (IBM Corp., Armonk, NY).

Results

First-generation students studied on campus more frequently than their counterparts (31% versus 20%, p < 0.05) and less at home in general (55.4% versus 67.5%, p < 0.05). Lower socioeconomic status (SES) students attended live Zoom lectures more often as well (56.6% versus 43.1%, p < 0.05). Lastly, no significant differences were found between disadvantaged and non-disadvantaged groups for the class of 2024 in the NBME exam scores or relationship quality scores.

Conclusion

Our results suggest that students from disadvantaged backgrounds spend more time studying on campus than at home. Additionally, during the COVID-19 pandemic, they attended live Zoom lectures more often than their non-disadvantaged counterparts. Access to campus was not restricted for KKSOM students during the pandemic. This may be one explanation for the lack of disparity between disadvantaged and non-disadvantaged students with regard to academic performance and relationship quality. This makes a strong argument for the importance of campus accessibility for the success of students, especially those from disadvantaged backgrounds.

## Introduction

Minority populations and patients from low socioeconomic backgrounds were disproportionately affected by the COVID-19 pandemic. Minority racial and ethnic groups had a higher incidence of COVID-19 infection, hospitalization, and death when compared to non-disadvantaged groups [1,2]. Furthermore, studies have shown that both adults and children who came from low-income neighborhoods had higher infection rates and frequently required life-saving measures such as invasive mechanical intubation [3,4].

Besides health outcomes, the COVID-19 pandemic also brought to light the challenges that students from disadvantaged backgrounds had to face when many schools and libraries closed due to social distancing. These students were taking advantage of school computers, Wi-Fi, printers, or just a quiet workspace, to which they suddenly did not have access. These resources are not often found in disadvantaged households, making it difficult for students to participate in distance learning. The disparities that disadvantaged groups face in technological hardware and software as well as their proficiency in it were amplified when students needed to transition to online learning [5,6].

A large technological disparity exists in the United States (US) between working-class families and their upper-/middle-class counterparts. About 41% of US working-class families do not have a laptop or desktop computer, and 43% do not have broadband access, whereas only 8% and 7% of upper- and middle-class US families do not, respectively [6]. Even if these working-class families have computers, they are often shared by multiple people in the household [6]. Many of these families do not have printers as well [6]. Hardware and software availability is only one aspect of this disparity. Working-class families are less likely to have dedicated work and study spaces available [6]. They are also more likely to have weak technological skills and less opportunity to develop these skills early on in life [6].

The setting of a global pandemic further limited the opportunity to interact, attend student affairs events, and develop strong social networks, which is a characteristic known to be correlated with student success [7]. Distance learning also impacted the ability of students to participate in faculty and peer mentoring, an important tool shown to positively affect academic outcomes in first-generation students [8]. These challenges that social distancing caused were especially apparent in the disadvantaged students taking care of dependents at home who already have baseline lower levels of student engagement [5,7]. A teacher in New York City with mostly Black and Hispanic students stated that “Many families have not been able to afford to continue to pay their Wi-Fi bill or have struggled to obtain internet capable devices” [5]. These students were also half as likely to interact with teachers in person, through video, or by phone [5]. Students of color were also found to engage less in distance learning and were more difficult to contact [5]. In other studies testing for mathematics, White students tested on average only one to three months behind where they were expected to be, whereas students of color tested three to five months behind [9]. Additionally, learning loss was measured to be 40% greater for students from working-class families than their upper-/middle-class family counterparts [6].

The impact of COVID-19 and school resource inaccessibility on underprivileged student education has been well established in the grade school setting but not at the university and graduate level. The study location tendencies of disadvantaged students during the pandemic may provide insight into the importance of campus availability and resource equality. This study aimed to analyze the frequency of different study locations used by medical students who are disadvantaged, underrepresented minorities (URM), first generation (first gen), or socioeconomically disadvantaged (SES disadvantaged) as compared to students who do not fit those characteristics throughout the pandemic. We also looked at their attendance of Zoom lectures during the pandemic. The subjects of our study, the students of Kirk Kerkorian School of Medicine (KKSOM) at the University of Nevada, Las Vegas (UNLV), had didactics and other on-campus activities transitioned from in-person to Zoom during the COVID-19 pandemic. The first-year students at the time, the class of 2024 (CO 2024), were most affected by this transition to online learning as they had the highest quantity of Zoom lectures and frequent exams. However, they always had access to campus and its resources throughout. Consequently, we hypothesized that during the pandemic, these disadvantaged groups were relatively protected or at least not affected in their academic and social outcomes as compared to their non-disadvantaged counterparts. Therefore, this study will also look at disadvantaged and non-disadvantaged students in the CO 2024 and the differences in their National Board of Medical Examiners (NBME) subject exam performance as well as the quality of their relationships with their faculty and fellow students during the pandemic to assess this hypothesis. The aim of this study is to gain insight into the inclination of disadvantaged students to study in different locations and to test our hypothesis of their equality in academic and social outcomes with continued campus availability during COVID-19 when compared to their non-disadvantaged counterparts.

This article was previously presented as a poster at the 2023 International Association of Medical Science Educators Annual Meeting, Cancun, Mexico, on June 11, 2023; a podium presentation at the 2023 Western Group Collaborative Spring Conference, Honolulu, Hawaii, on April 16, 2023; and as a poster at the 2023 Clark County Medical Society Research Symposium, Las Vegas, NV, on January 28, 2023. This article was previously posted to the Research Square preprint server on May 24, 2023.

## Materials and methods

Our study was conducted during the 2020-21 academic year. A de-identified database of program evaluation data was provided in accordance with the approved IRB protocol (#1030906-1), titled "School of Medicine use of program evaluation data for research." The database included demographic information and survey data regarding study location usage, live Zoom lecture attendance, student/faculty, and student/student relationship quality during the pandemic. The subjects of the study were first-, second-, third-, and fourth-year students of KKSOM at the UNLV. The class of 2024 (CO 2024) had a unique experience as they were first-year students during the COVID-19 pandemic and were transitioning to online learning. However, they always had access to campus and technological resources. Although all students had optional Zoom lectures and exams during the pandemic, first-year students had them much more often due to the natural course of the medical school curriculum. A questionnaire was formulated for the study. Steps were taken to ensure standardization, such as the selection of target respondents and methods utilized to reach respondents, appropriate wording and sequencing of the questionnaire items, and reliability trial (Cronbach's alpha = 0.7734) as well as face and content validity prior to administration. Response rates were 48/58 for the first-year students (CO 2024), 45/60 for the second-year students, 33/60 for the third-year students, and 41/60 for the fourth-year students.

Study location questions asked about the percentage of time students studied at home, on campus, or elsewhere during the pandemic. Students in the CO 2024 were asked about their relationships during the pandemic. Student/faculty relationships and student/student relationships used a five-point scale from 1-5, with 1 being “not very well” and 5 being “very well.” Student/faculty relationships asked, “How well do you feel like you got to know your faculty during that year?,” and student/student relationships asked, “How well do you feel like you got to know your classmates during that year?.” Incomplete responses or responses with missing data were removed from the aggregate prior to data analysis. These survey items are expected to incorporate a degree of bias in recorded responses. The preference for providing socially favorable responses to these questions represents an inherent degree of social-desirability bias or agreement bias. We aimed to mitigate the effect of this by ensuring and emphasizing the anonymity of survey responses. Distributing a survey to a given population can also involve unintentional sampling bias. This effect was minimized by sharing the link to the survey with all students' school-verified emails from the principal investigator.

Data analysis of these factors compared students who were disadvantaged, underrepresented minority, first-generation, or socioeconomically disadvantaged and students who did not fit those characteristics. We refer to these groups of students collectively as “disadvantaged students” throughout our study. Disadvantaged status, underrepresented minority, and first-generation were self-designated by the students themselves in the survey. We classified students as socioeconomically disadvantaged using the Association of American Medical Colleges (AAMC) socioeconomic status (SES) indicator, which classifies students as economic/occupation (EO) 1-5. These categories are defined by the student’s parental education and occupation. Students who were indicated as EO1 or EO2 were marked as socioeconomically disadvantaged. EO1 is indicated when the student’s parental education is less than a bachelor’s degree and EO2 when their parental occupation falls under service, clerical, skilled, or unskilled labor categories at any educational level. To assess whether keeping the campus and resources accessible during the pandemic may have contributed to their academic success, we also analyzed NBME subject exam score averages between the different demographic groups of CO 2024 and their counterparts. To assess if the continued access also contributed to social success, we analyzed the relationship quality of the groups in CO 2024. The analysis was completed using independent samples t-tests and Cronbach’s alpha through SPSS Statistics software version 28.0.1.1 (IBM Corp., Armonk, NY).

## Results

We first sought to determine how each of our eight groups allotted their study time between three location designations: home, campus, or elsewhere. Every respondent provided their percentage of total study time between these locations; for each location, percentages were averaged, and standard deviations (SDs) were calculated. Additionally, a Pearson’s unpaired t-test was performed between both groups (non-disadvantaged and disadvantaged) within each of the three disadvantage statuses. The results of this investigation are shown in Tables 1-3. The group of respondents (Group), number of respondents (N), the average taken from all the respondents' answers (Mean), SD, and the one-sided p-value are shown in each table. Each table also denotes an asterisk (*) next to p-values < 0.05. Table 1 indicates the time each group spent studying at home.

**Table 1:** Percentage of time studying at home for each group *p-values < 0.05. URM: Underrepresented minorities; SES: Socioeconomic status.

Group	N	Mean	SD	One-sided p-value
Non-URM	138	65.30%	31.80%	0.146
URM	29	58.20%	37.30%	
Non-first gen	125	67.50%	30.40%	*0.029
First gen	42	55.40%	36.60%	
Non-disadvantaged	135	66.40%	30.60%	0.087
Disadvantaged	32	56.30%	38.60%	
Non-SES disadvantaged	114	65.70%	31.70%	0.18
SES disadvantaged	53	60.70%	34.90%	

First-generation students studied at home on average 12.14% less than their counterparts, which is a statistically significant difference. Disadvantaged students were also found to study at home on average 9.63% less.

Table 2 indicates the time each group spent studying on campus.

**Table 2:** Percentage of time studying on campus for each group *p-values < 0.05. URM: Underrepresented minorities; SES: Socioeconomic status.

Group	N	Mean	SD	One-sided p-value
Non-URM	138	21.5%	24.2%	0.082
URM	29	30.7%	31.8%	
Non-first gen	125	20%	22.8%	*0.021
First gen	42	31%	31.5%	
Non-disadvantaged	135	21.4%	23.9%	0.112
Disadvantaged	32	28.7%	31.7%	
Non-SES disadvantaged	114	21.8%	24.6%	0.206
SES disadvantaged	53	25.4%	28%	

First-generation students studied 10.79% more on campus than their counterparts. Underrepresented minority students and disadvantaged students studied 9.21% (p = 0.082) and 8.28% (p = 0.090) more than their counterparts on campus, respectively, though this finding is not statistically significant.

Table 3 indicates the time each group spent studying not at home or on campus but elsewhere.

**Table 3:** Percentage of time studying elsewhere for each group *p-values < 0.05. URM: Underrepresented minorities; SES: Socioeconomic status.

Group	N	Mean	SD	One-sided p-value
Non-URM	138	11.3%	18.7%	0.308
URM	29	13.2%	22.1%	
Non-first gen	125	11.7%	19.8%	0.469
First gen	42	11.4%	17.9%	
Non-disadvantaged	135	11.5%	19%	0.452
Disadvantaged	32	12%	20.6%	
Non-SES disadvantaged	114	10.1%	18.5%	0.066
SES disadvantaged	53	14.9%	20.6%	

Though this finding is not statistically significant, it appears that socioeconomically disadvantaged students trended toward studying elsewhere (4.98%) more than their counterparts.

We then sought to determine how each of the eight groups compared in live Zoom lecture attendance. Each respondent was queried regarding the frequency of attendance. Sixteen respondents surveyed did not answer this question. Responses within each group were averaged, and SDs were calculated. Additionally, a Pearson’s unpaired t-test was performed between both groups (non-disadvantaged and disadvantaged) within each of the three disadvantage statuses. The results of this investigation are shown in Table 4.

**Table 4:** Percentage of live Zoom lectures attended by each group *p-values < 0.05. URM: Underrepresented minorities; SES: Socioeconomic status.

Group	N	Mean	SD	One-sided p-value
Non-URM	122	45.9%	36.1%	0.116
URM	29	56.6%	43.1%	
Non-first gen	115	47%	37%	0.377
First gen	36	49.3%	40.3%	
Non-disadvantaged	124	47.5%	36.8%	0.487
Disadvantaged	27	47.8%	42.3%	
Non-SES disadvantaged	101	43.1%	37.2%	*0.019
SES disadvantaged	50	56.6%	37.4%	

Our results suggest that socioeconomically disadvantaged students attended live Zoom lectures (13.55%) more than their counterparts. No significant difference in lecture attendance was noted between the URM, first-generation, or disadvantaged groups. 

Next, we aimed to determine the NBME exam scores of each of the eight groups. Each student’s exam score was averaged, and then these averages were combined for each respective group and averaged again to determine a representative mean NBME score with SDs. A Pearson’s unpaired t-test was performed between both groups (non-disadvantaged and disadvantaged) within each of the four disadvantaged statuses to look for differences in performance. The results are shown in Table 5.

**Table 5:** The mean NBME scores of each group in the CO 2024 *p-values < 0.05. URM: Underrepresented minorities; SES: Socioeconomic status; CO 2024: Class of 2024; NBME: National Board of Medical Examiners.

Group	N	Mean	SD	One-sided p-value
Non-URM	41	84.6%	5.1%	0.485
URM	7	84.7%	5.7%	
Non-first gen	38	84.9%	5.5%	0.259
First gen	10	83.7%	3.8%	
Non-disadvantaged	38	84.7%	5.4%	0.460
Disadvantaged	10	84.5%	4.4%	
Non-SES disadvantaged	36	84.7%	5.1%	0.455
SES disadvantaged	12	84.5%	5.4%	

There were no distinguishable differences in the NBME average exam scores between each of the demographic groups and their counterparts. 

Next, we sought to analyze the quality of student-student relationships within this class. Each student’s response, an integer between 1 and 5, was averaged within their respective group to provide means and SDs. Additionally, a Pearson’s unpaired t-test was performed between both groups (non-disadvantaged and disadvantaged) within each of the three disadvantage statuses. The results are shown in Table 6.

**Table 6:** Quality of student-student relationships for the CO 2024 rated 1-5 *p-values < 0.05. URM: Underrepresented minorities; SES: Socioeconomic status; CO 2024: Class of 2024.

Group	N	Mean	SD	One-sided p-value
Non-URM	41	2.37	.799	0.253
URM	7	2.14	.900	
Non-first gen	38	2.37	.819	0.282
First gen	10	2.20	.789	
Non-disadvantaged	38	2.29	.802	0.235
Disadvantaged	10	2.50	.850	
Non-SES disadvantaged	36	2.33	.828	0.500
SES disadvantaged	12	2.33	.778	

There were no significant differences in the quality of student-student relationships between the groups.

In a similar manner, we analyzed the quality of student-faculty relationships. Each student’s response was averaged within their respective group to provide means and SDs. Additionally, a Pearson’s unpaired t-test was performed between both groups (non-disadvantaged and disadvantaged) within each of the three disadvantage statuses. The results are shown in Table 7.

**Table 7:** Quality of student-faculty relationships of CO 2024 rated 1-5 *p-values < 0.05. URM: Underrepresented minorities; SES: Socioeconomic status; CO 2024: Class of 2024.

Group	N	Mean	SD	One-sided p-value
Non-URM	41	1.76	.767	0.377
URM	7	1.86	.900	
Non-first gen	38	1.82	.692	0.221
First gen	10	1.60	1.075	
Non-disadvantaged	38	1.84	.789	0.110
Disadvantaged	10	1.50	.707	
Non-SES disadvantaged	36	1.83	.811	0.170
SES disadvantaged	12	1.58	.669	

There were no significant differences in the quality of student-faculty relationships between the groups.

## Discussion

These results indicate that various disadvantaged groups of students may utilize campus and its resources more often than their counterparts and study at home less often. There could be many reasons for this finding. Disparity in hardware and software availability and quality, lack of a proper home workspace, and unstable living conditions among disadvantaged students are all possible factors [5,7]. Access to a computer and internet connection are positively correlated to the extent of parental schooling, occupation, and income [10]. About 90% of high-SES households have Internet access, while as little as 40% of low-SES households have Internet access [10]. The importance of this accessibility cannot be overstated. Lei et al. found that students who spent up to three hours of their day on computers had an overall higher GPA [11]. This is usually only possible with an at-home computer. Even with access to a home computer, working-class parents are less likely to direct their children’s computer use toward educational endeavors, partly due to their own lack of technology use [12].

High-SES households are also more likely to consistently use information technology (IT) services and therefore develop and maintain technological skills [10]. A student’s technological competence has a substantial impact on their self-efficacy as it pertains to online school work [13]. The independent and isolated nature of online learning is an arduous test of a student’s self-efficacy, which is defined as one’s confidence in their ability to complete performance tasks [13,14]. School campus offers a remedy for disadvantaged students who may have trouble accessing IT services, share a computer at home, have a poor internet connection, and/or lack a quiet workspace. The campus also offers solitude from a hectic home environment and allows for heightened student and faculty engagement, which is correlated with greater academic success [8]. These differences make disadvantaged students more reliant on campus resources than their counterparts and therefore potentially more affected by school and library closings.

If there comes a time again when social distancing and closure of schools and libraries are necessary, there must be an effort to provide students with continued access to campus resources. Explicit and clear guides to online coursework, accessible IT services, and continuous communication between teacher and student are required to bridge the gap between digital literacy levels and academic success. Schools and governments must ensure students still have access to computers, Wi-Fi, and dedicated study spaces. In Australia, for example, the government is proposing to rent out computers to all secondary students and ensure high-speed internet access to 98% of all homes [15]. The English government has made the same commitment to all high school and college students so they can access their school work [16].

This study does have several limitations. First, data was collected from a single medical school that has a student demographic that may not reflect the general population. This limits the generalizability of these findings to other institutions. Second, the survey responses are subject to self-reporting bias. Lastly, although our survey assessed where students were studying during the COVID-19 pandemic, it did not specifically ask respondents about their access to resources outside of the university setting and if this lack of resources was what specifically impacted their education. Another limitation is that the students who fit these demographic categories were not compared to students who did not fit any of the other demographic characteristics. The fact that some students being compared fit into another disadvantaged group may have reduced the power of our statistical outputs.

## Conclusions

Disadvantaged students may spend more time studying on campus, less time studying at home, and rely more on live classroom lectures. The students from KKSOM at UNLV had no lapse in their ability to access campus and resources throughout the COVID-19 pandemic. This continued accessibility may have provided the needed support to allow the disadvantaged groups to have no significant differences compared to their counterparts in NBME mean exam scores or their relationship quality with fellow students and faculty. This finding makes a strong argument for the importance of campus accessibility and the accompanying resources for the overall success of students, especially those who come from disadvantaged backgrounds. In the event of a global pandemic or a shift to online learning, school administration should take extra efforts to ensure that students have access to any educational resources, technologies, or study spaces that they may need. An important pillar of equality of opportunity and academic success for disadvantaged student populations is scholastic resource accessibility and management.
